# Correlation between lumbar intervertebral disc height and lumbar spine sagittal alignment among asymptomatic Asian young adults

**DOI:** 10.1186/s13018-018-0737-x

**Published:** 2018-02-12

**Authors:** Feng Zhang, Kai Zhang, Hai-Jun Tian, Ai-Min Wu, Xiao-Fei Cheng, Tang-Jun Zhou, Jie Zhao

**Affiliations:** Ninth People’s Hospital, Department of Orthopaedics, Shanghai Key Laboratory of Orthopaedic Implants, Shanghai Jiaotong University School of Medicine, 639 Zhizaoju Road, Shanghai, People’s Republic of China

**Keywords:** Intervertebral disc height, Pelvic incidence, Lumbar spine sagittal alignment, Roussouly classification

## Abstract

**Background:**

To investigate the distribution and characteristics of the lumbar intervertebral disc height (IDH) in asymptomatic Asian population and to determine whether the lumbar IDH is related to the lumbar spine sagittal alignment.

**Methods:**

A cohort of 169 cases of asymptomatic volunteers was enrolled from January 2014 to July 2016. All participants underwent magnetic resonance imaging of the lumbar spine and panoramic radiography of the spine. Panoramic radiographs of the spine were taken to evaluate pelvic incidence (PI), sacral slope (SS), and pelvic tilt (PT) using Surgimap® software. Roussouly classification was utilized to categorize all subjects according to the four subtypes of sagittal alignment. The IDH was measured on the MRI mid-saggital section of the vertebral body. The relationships between lumbar IDH and spine-pelvic parameters were also assessed using the Spearman correlation analysis.

**Results:**

The reference value ranges of IDH in asymptomatic Asian volunteers between L1/2, L2/3, L3/4, L4/5, and L5/S1 were (6.25, 10.99), (6.97, 12.08), (7.42, 13.3), (7.76, 14.57),and (7.11, 13.12) mm, respectively. Based on the above reference value, the high lumbar intervertebral space is defined as more than 14 mm. According to the Roussouly Classification, there are 33 cases in type I, 48 in type II, 66 in type III, and 22 in type IV. According to the definition of the high IDH, there are two cases in type I, three in type II, nine in type III, and eight in type IV. The results indicated that people in the Roussouly III and IV subtypes had greater values for IDH compared to those of Roussouly I and II subtypes, and the spinopelvic parameters were partly correlated with IDH in different subtypes. In addition, levels L4–L5 showed the highest IDH for all four groups followed by the L3–L4 and L5–S1 levels, and the value of L3–L4 is equivalent to that of L5–S1. All type groups showed moderate and positive correlations between the PI and IDH except the level of L1–L2 in type IV.

**Conclusions:**

The IDH may influence the lumbar spine sagittal alignment in asymptomatic Asian adults. Moreover, pre-operative evaluation of IDH is useful for selection of optimal cage size and reconstruction of spinal alignment.

## Background

The reduction of lumbar intervertebral disc height (IDH) is the key point in the pathological process of intervertebral disc degeneration (IVDD), and the diseases of lumbar degeneration often demonstrate the reduction of intervertebral disc height and the change of lumbar spine sagittal alignment in the radiographic images. For the treatment of degenerative lumbar diseases, lumbar interbody fusion is now widely used. During operation, the restoration of the lumbar sagittal alignment by using appropriate interbody fusion cage is meaningful, considering the segment between L4 and S1 contributes approximately 60 to 80% of lumbar lordosis [1].

However, it is arduous to determine the reasonable intervertebral disc height because of the individual difference of IDH and the lack of anatomical parameters.

It has been manifested according to anatomical measurements that the total IDH is about 25% of the total length of the spine and the IDH of lumbar is about 30–36% of the length of lumbar [2]. Much of the research has examined that the morphology of intervertebral space has a pivotal role in the lumbar spine sagittal alignment and the intervertebral space is closely related to the type and motion of lumbar spine. A number of studies have focused on the importance of the spinal balance and curvature in the normal function and in various disease states [3, 4]. Roussouly et al. proposed a classification system with four postural subtypes based on the sagittal balance patterns observed in asymptomatic young adults. This classification is based on SS grade and vertebral curvatures. It is known that the reciprocal association exists between the sacral slope and pelvic incidence and the types of sagittal alignment in asymptomatic young adults [5]. However, little is known about the distribution characteristics of the height of intervertebral space and no study has investigated the relationship between IDH and these various subtypes of sagittal alignment in asymptomatic young adults.

The judgment of normality can be possible by analyzing the normal characteristics of intervertebral disc height in the different patterns of sagittal curvature. The abnormal intervertebral disc height should be restored to normal state on the basis of the original height. In addition, the amount of intervertebral disc height for restoration of normal sagittal curvatures is related with the original patterns of sagittal curvatures.

In order to address these deficits, we sought to provide normative parameters of IDH and the relationship between IDH and lumbar spine sagittal alignment in a series of asymptomatic young adults. We sought to provide spinal surgeons with values they might use as a reference when planning lumbar interbody fusion.

## Methods

### Subjects

**Fig. 1:**
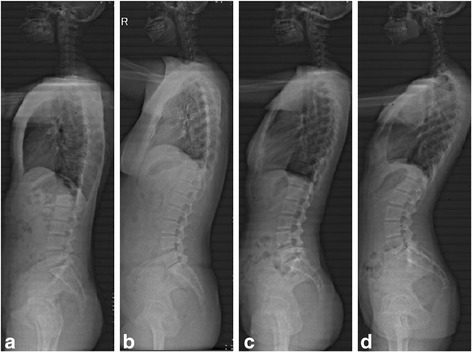
Panoramic radiographic of four types of sagittal alignment. **a** Roussouly type I. **b** Roussouly type II. **c** Roussouly type III. **d** Roussouly type IV

A cohort of 169 asymptomatic Asian subjects (including 72 males and 97 females, average age 25.5 ± 5.6 years old) were recruited prospectively between March 2012 and May 2015. Exclusion criteria established for the study were, prior spinal surgery or instrumentation, previous lumbar pain (described in medical files), significant degeneration (disc height collapse), spinal deformity, osteoporosis, and modic change. The height and body mass index (BMI) of all subjects were measured. The participants were divided into four groups according to the lumbar spine subtype classification of Roussouly [5] based on a panoramic radiography evaluation (Fig. 1): type I: *n* = 33, type II: *n* = 48, type III: *n* = 66, and type IV: *n* = 22.

All volunteers provided informed consent. The ethical permission was given by the university as a retrospective study.

For the sake of reducing deviation and consistency, two senior spine surgeons conducted the measurement using the picture archiving communication system (PACS) workstations (a picture analyzing system, Shanghai Jiaotong University School of Medicine), respectively. If they disagreed, a third one was invited to make a final decision.

### Magnetic resonance imaging (MRI)

A 1.5 Tesla magnet using the spine array coil (Siemens, Erlangen, Germany) was used for the MRI. In the sagittal planes, the T1-weigheted images were constructed with a repetition time (TR) of 500 ms and echo time (TE) of 15 ms with three acquisitions and two saturations of the lumbosacral spine showing L1 to S1, comprising five consecutive images for each patient. Images were taken using 4-mm slice thickness and measurements were performed on the T1 intensity images.

**Fig. 2:**
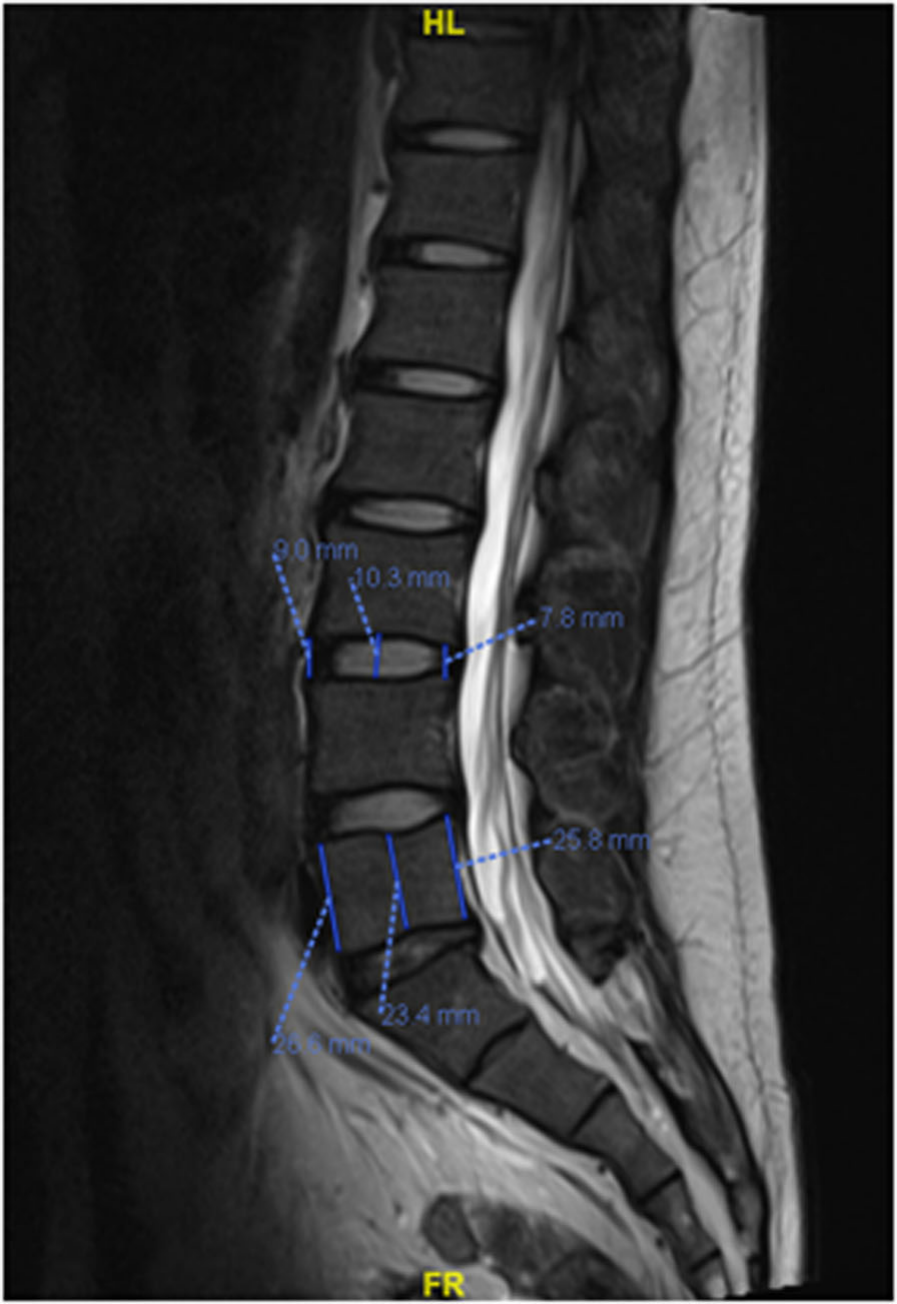
IDH and VH measured on the mid-saggital section of the vertebral body using the PACS workstation

The height of the intervertebral space was measured on the mid-saggital section of the vertebral body, as seen on MRI. On the best image, the intervertebral disc height was digitized by electronic cursor and was labeled. Using the measurement tool of the PACS workstation, the front, middle, and rear heights of the intervertebral spaces of L1/2, L2/3, L3/4, L4/5, and L5/S1 were determined on the computer as shown in Fig. 2. The height of the intervertebral space was obtained by (front height + middle height + rear height of intervertebral space)/3. The vertebral body height (VH) was obtained by (height of anterior wall + height of middle wall + height of posterior wall of vertebral body)/3. Then, the ratio of IDH/VH was obtained.

### Panoramic radiography

The radiological examination protocol was standardized for all participants. For each participant, standing anteroposterior and left lateral radiographs covering the spine and the pelvis were obtained. Subjects were instructed to stand in a comfortable position, with the hips and knees fully extended. The arms were flexed with the hands rested on supports at the level of their shoulders [6]. All radiographs were obtained in digital format. Parameters related to sagittal alignments were then measured with Surgimap (Spine Software, version 1.1.2, NY).

**Fig. 3:**
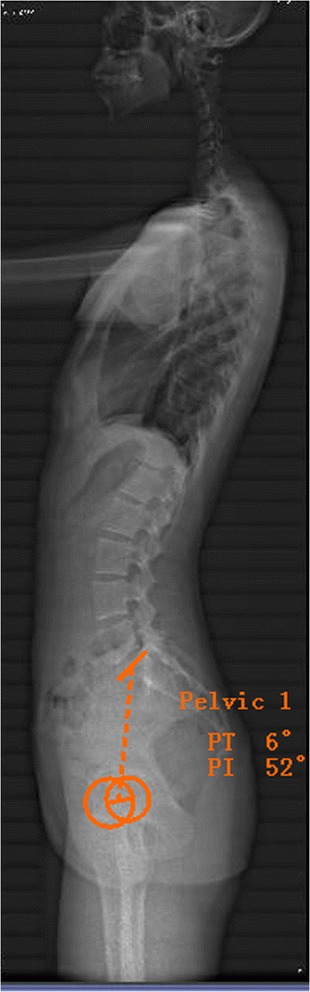
Pelvic parameters measured with Surgimap: pelvic tilt (PT) and pelvic incident (PI)

The global radiological parameters (Fig. 3) included the pelvic incidence (PI, the angle between a line drawn from center of the hip axis to the center of the superior endplate of S1 and perpendicular to the endplate), sacral slope (SS, the angle subtended by the horizontal line and upper sacral endplate), pelvic tilt (PT, the angle between the vertical plane and a straight line joining the centers of the femoral heads and the center of the superior endplate of S1), and local lordosis of each intervertebral disc space (LL, the angle subtended by lower endplate of upper vertebral body and upper endplate of lower vertebral body) [7].

### Statistical analyses

All statistical analyses were performed with the SPSS 15.0 software (SPSS Inc., Chicago, IL, USA). Statistical significance was set at 0.05. Descriptive statistics in the form of mean ± SD for all spine parameters were provided for all subjects. Each reference interval represents the central 95% of the sample distribution and the reference value range for IDH was obtained by mean ± 1.96 s.

The one-way ANOVA test and Student *t* tests for independent samples were also utilized to evaluate the parameters among different levels and groups. Spearman’s correlation was used to verify the correlations between IDH and the spinopelvic parameters of the sample.

Interobserver analyses for the parameters were assessed using intraclass correlation coefficient (ICC).

## Results

Tables 1, 2, and 3 show the distribution of the height and lordosis of intervertebral space and the ratio of IDH/VH in each vertebral segment (through L1–L2 to L5–S1) for each of the four Roussouly subtypes. Level L4–L5 showed the highest IDH for all four groups, followed by the L3–L4 and L5–S1 levels. And, level L1–L2 demonstrated the lowest IDH for all. In types I, II, and III, there was significant statistical difference between them except between the level of L5–S1 and L3–L4 and L2–L3. In type IV, there was significant statistical difference between the level of L1–L2 and L3–L4, L4–L5, and L5–S1, and between the level of L2–L3 and L4–L5, and between the level of L4–L5 and L5–S1. And, there was no statistical difference between the rest.

**Table 1:** Distribution of IDH in each vertebral segment (through L1–L2 to L5–S1) for the four Roussouly subtypes

IDH	L1/2	L2/3	L3/4	L4/5	L5/S1
Type I	8.276 ± 0.8697	9.063 ± 0.8253	9.814 ± 0.9908	10.56 ± 1.164	9.696 ± 1.138
Type II	8.094 ± 1.123	8.904 ± 1.139	9.688 ± 1.215	10.42 ± 1.446	9.556 ± 1.313
Type III	8.864 ± 1.257	9.848 ± 1.361	10.80 ± 1.607	11.63 ± 1.903	10.55 ± 1.558
Type IV	9.584 ± 0.9308	10.55 ± 1.128	11.34 ± 1.423	12.31 ± 1.511	10.96 ± 1.875

**Table 2:** Distribution of the Lordosis of each intervertebral disc space in the four Roussouly subtypes

IDH	L1/2	L2/3	L3/4	L4/5	L5/S1
Type I	3.758 ± 2.606	6.455 ± 2.548	8.212 ± 3.072	13.273 ± 3.824	10.424 ± 4.221
Type II	3.354 ± 2.586	6.562 ± 2.915	8.875 ± 3.509	13.229 ± 4.283	11.083 ± 4.877
Type III	3.955 ± 2.567	7.015 ± 2.602	9.939 ± 2.763	14.167 ± 3.595	11.560 ± 4.412
Type IV	4.727 ± 2.926	8.045 ± 3.022	12.045 ± 1.551	16.091 ± 3.218	15.136 ± 3.442

**Table 3:** Distribution of the ratio of IDH/VH in each vertebral segment (through L1–L2 to L5–S1) for the four Roussouly subtypes

IDH/VH(%)	L1/2	L2/3	L3/4	L4/5	L5/S1
Type I	39.98 ± 5.35	42.84 ± 4.1	45.09 ± 4.11	47.14 ± 3.65	43.00 ± 5.23
Type II	37.84 ± 5.79	40.46 ± 5.13	43.01 ± 4.70	45.59 ± 4.71	41.15 ± 5.14
Type III	39.08 ± 5.54	42.31 ± 5.26	45.20 ± 5.25	47.64 ± 5.03	43.16 ± 4.94
Type IV	43.17 ± 3.84	45.57 ± 3.15	47.02 ± 3.67	49.9 ± 3.16	44.13 ± 5.92

Among a total of 169 volunteers, the reference value range for IDH from L1–L2 to L5–S1 was 8.62 ± 1.21 (6.25, 10.99), 9.52 ± 1.30 (6.97, 12.08), 10.36 ± 1.50 (7.42, 13.30), 11.17 ± 1.74 (7.76, 14.57), and 10.15 ± 1.55 (7.11, 13.12) mm. Based on the reference value, it was suggested that the tall lumbar intervertebral disc height was defined as more than 14 mm. According to the definition, there were two cases with tall lumbar intervertebral space in type I, three cases in type II, 13 cases in type III, and eight cases in type IV.

**Fig. 4:**
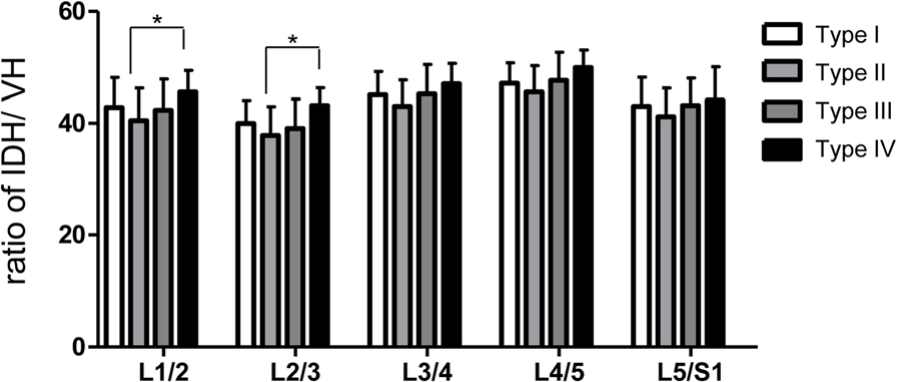
The ratio of IDH/VH in the same segment intercompared in the four subtypes. There was significant statistical difference between types II vs. IV in the level of L1–L2 and L2–L3

Figure 4 shows the ratio of IDH/VH in the same segment intercompared in the four subtypes. There was significant statistical difference between types II vs. IV in the level of L1–L2 and L2–L3.

As showed in Table 4, significant differences were found between the IDH of different Roussouly types in asymptomatic subjects. In the levels of L2–L3, L3–L4, L4–L5, and L5–S1, there was significant statistical difference between them except type I vs. type II and types III vs. IV. However, in the level of L1–L2, there was no statistical difference between type I and type II, and type III.

**Table 4:** Difference between the IDH of different Roussouly types in asymptomatic subjects

	L1/2		L2/3		L3/4		L4/5		L5/S1	
Type	Mean Diff.	*P* value	Mean Diff.	*P* value	Mean Diff.	*P* value	Mean Diff	*P* value	Mean Diff.	*P* value
I x II	0.1826	0.8867	0.1598	0.9324	0.1259	0.9774	0.1322	0.9834	0.1399	0.9746
I x III	− 0.5873	0.0676	− 0.7850	0.0114*	− 0.9876	0.0051*	− 1.071	0.0110*	− 0.8494	0.0361*
I x IV	− 1.307	0.0002*	− 1.484	< 0.0001*	− 1.525	0.0005*	− 1.754	0.0006*	− 1.262	0.0111*
II x III	− 0.7699	0.0020*	− 0.9448	0.0002*	− 1.114	0.0002*	− 1.203	0.0007*	− 0.9893	0.0027*
II x IV	− 1.490	< 0.0001*	− 1.644	< 0.0001*	− 1.651	< 0.0001*	− 1.886	< 0.0001*	− 1.401	0.0016*
III x IV	− 0.7200	0.0460*	− 0.6989	0.0800	− 0.5374	0.3871	− 0.6833	0.3117	− 0.4121	0.6636

*There was a statistically significant difference (*p* <  0.05)

Table 5 shows the correlations (*r*) between IDH and PI for each group. All type groups showed moderate and positive correlations between the PI and IDH; only the level of L1–L2 in type IV was not correlated with PI.

**Table 5:** Correlations (*r*) between IDH and PI for each group

Roussouly types	Level	*r*	*p*
Type I	L1–L2	0.4266	0.0133
	L2–L3	0.3992	0.0214
	L3–L4	0.5033	0.0028
	L4–L5	0.4008	0.0208
	L5–S1	0.4855	0.0042
Type II	L1–L2	0.4429	0.0016
	L2–L3	0.4907	0.0004
	L3–L4	0.5081	0.0002
	L4–L5	0.4960	0.0003
	L5–S1	0.4360	0.0020
Type III	L1–L2	0.4989	< 0.0001
	L2–L3	0.5396	< 0.0001
	L3–L4	0.4683	< 0.0001
	L4–L5	0.5928	< 0.0001
	L5–S1	0.5203	< 0.0001
Type IV	L1–L2	0.4220	0.0504*
	L2–L3	0.5656	0.0061
	L3–L4	0.7636	< 0.000
	L4–L5	0.7645	< 0.000
	L5–S1	0.6438	0.0012

*There was no statistically significant correlation (*p* > 0.05)

Table 6 shows the correlations (*r*) between Lordosis and PI for each group. All type groups showed moderate and positive correlations between the PI and Lordosis; only the level of L1–L2 in types I and IV was not correlated with PI.

**Table 6:** Correlations (*r*) between Lordosis and PI for each group

Roussouly types	Level	*r*	*p*
Type I	L1–L2	0.4034	0.0521*
	L2–L3	0.4183	0.0325
	L3–L4	0.4978	0.0032
	L4–L5	0.5027	0.0195
	L5–S1	0.5321	< 0.0001
Type II	L1–L2	0.4327	0.0027
	L2–L3	0.4856	0.0019
	L3–L4	0.5154	0.0002
	L4–L5	0.5360	0.0002
	L5–S1	0.4859	0.0018
Type III	L1–L2	0.5017	< 0.0001
	L2–L3	0.5483	< 0.0001
	L3–L4	0.4892	< 0.0001
	L4–L5	0.6098	< 0.0001
	L5–S1	0.5709	< 0.0001
Type IV	L1–L2	0.4311	0.0527*
	L2–L3	0.5828	0.0078
	L3–L4	0.8619	< 0.0001
	L4–L5	0.8456	< 0.0001
	L5–S1	0.7389	< 0.0001

*There was no statistically significant correlation (*p* > 0.05)

Among all volunteers, the reference value range for height from type I to type IV was 170.55 ± 4.21, 171.01 ± 3.28, 169.77 ± 4.50, and 171.17 ± 2.74 cm. And, the value of BMI from type I to type IV was 19.66 ± 0.31, 20.21 ± 0.48, 19.87 ± 0.50, and 21.17 ± 0.74. In the height and BMI, there was no statistical difference between type I, type II, type III, and type IV.

The ICCs for all parameters were considered as excellent (L1/2: ICC = 0.94, 95% CIs = 0.89–0.97; L2/3: ICC = 0.87, 95% CIs = 0.69–0.97; L3/4: ICC = 0.90, 95% CIs = 0.84–0.95; L4/5: ICC = 0.92, 95% CIs = 0.86–0.95; L5/S1 = 0.89, 95% CIs = 0.82–0.92).

## Discussion

Previous studies have focused on lumbar spine sagittal alignment individually or have only included patients with back pain and disc degeneration [8–14]. However, no research has directly evaluated the association between sagittal alignment subtype and IDH in asymptomatic young adults. We classified a sample of asymptomatic young adults based on the four subtypes of sagittal alignment proposed by Roussouly, and we examined whether any of these subtypes had a relationship with IDH at each disc level. The current study revealed that people in Roussouly III and IV subtypes had greater values for IDH compared to those of Roussouly I and II subtypes, and the spinopelvic parameters were partly correlated with the height of intervertebral space in different subtypes. In addition, level L4–L5 showed the highest IDH for all four groups followed by the L3–L4 and L5–S1 levels, and the value of L3–L4 is equivalent to that of L5–S1.

The results of our previous studies indicate that the cage of relatively larger size should be appropriately placed into the disc space to improve the stability and the spinal alignment [15–17]. But, it is very difficult to select the appropriate size of cage. Present results will help to partly solve this problem.

The population of tall lumbar intervertebral space occupies a certain proportion in four subtypes in asymptomatic adults. The ratio of tall intervertebral space in types III and IV was greater than in types I and II significantly. The Roussouly I and II subtypes are characterized by mild lumbar curvatures and an SS of less than 35°. These characteristics are different from those of III and IV subtypes, which have well-defined curvatures and high SS values [18]. When the lumbar spine is hypolordotic and flat, contact forces primarily act on the anterior region of the spine, vertebral bodies, and discs, thereby increasing disc pressure [13, 18, 19]. Because of these characteristics, the pressure of axial load transfer through the lumbar disc in Roussouly I and II subtypes is greater than in types III and IV. Disc space changes, especially increase in disc space height in response to reduced axial loads, were documented in a few previous studies. Then, the greater pressure may be one of the reasons for the low IDH in types I and II.

The degenerative disc diseases in lumbar spine frequently show the reduction of IDH, and lumbar interbody fusion is now widely accepted by surgeons for these diseases. For the case of spine fusion, the intervertebral segment fusion rate and the intervertebral foramen height are paramount, and these factors are associated with the height of instruments such as the cage inserted to the intervertebral disc space. Nevertheless, the instruments that have been currently used are prepared to fit to the size of the intervertebral space of Caucasians, and thus, it may not fit the smaller Asians. Consequently, in our research, the intervertebral disc height of Chinese was measured by performing MRI. The study demonstrated that in types I and II, the value of IDH and PI was lower than in types III and IV. Accordingly, during the lumbar interbody fusion for cases in types I and II, it is important to reconstruct the height of intervertebral space by using a cage without excessive correction of lumbar lordosis. In contrast, during interbody fusion for cases in types III and IV, it is crucial to restore not only the height of IDH but also lumbar lordosis.

One clinically relevant finding of our study was that the spinopelvic parameters were associated with the height of intervertebral space in all subtypes except the level of L1–L2 in type IV. It is meaningful to predict the IDH of the degenerative segment. In the research, because the IDH of the L4–L5 level was significantly taller than that of the other levels, it is faulty to evaluate the IDH of the L4–L5 level through the adjacent segment. It is beneficial to predict the IDH of the degenerative segment with the value of PI.

As for the limitation of this article, our study was cross-sectional; and a future longitudinal study investigating how spine sagittal alignment and IDH influence each other over time would be valuable.

## Conclusion

The population of high lumbar intervertebral space occupies a certain proportion in asymptomatic Asian population. The IDH may influence the lumbar spine sagittal alignment. Evaluation of height of intervertebral space of the degenerative segment with the value of PI before operation is useful for selection of lumbar fusion cage specifications, restoration IDH, and reconstruction lumbar spine sagittal alignment.

## Abbreviations

ICC: Interclass correlation coefficient
IDH: Intervertebral disc height
IVDD: Intervertebral disc degeneration
L1: The first lumbar
L2: The second lumbar
L3: The third lumbar
L4: The forth lumbar
L5: The fifth lumbar
MRI: Magnetic resonance imaging
PI: Pelvic incidence
PT: Pelvic tilt
S1: The first sacral
SS: Sacral slope
TE: Echo time
TR: Repetition time
VH: Vertebral body height
